# The Influence of Efflux Pump Inhibitors on the Activity of Non-Antibiotic NSAIDS against Gram-Negative Rods

**DOI:** 10.1371/journal.pone.0147131

**Published:** 2016-01-15

**Authors:** Agnieszka E. Laudy, Agnieszka Mrowka, Joanna Krajewska, Stefan Tyski

**Affiliations:** 1 Department of Pharmaceutical Microbiology, Medical University of Warsaw, Oczki 3 Str., 02-007, Warsaw, Poland; 2 Department of Antibiotics and Microbiology, National Medicines Institute, Chełmska 30/34 Str., 00-725, Warsaw, Poland; University of Technology Sydney, AUSTRALIA

## Abstract

**Background:**

Most patients with bacterial infections suffer from fever and various pains that require complex treatments with antibiotics, antipyretics, and analgaesics. The most common drugs used to relieve these symptoms are non-steroidal anti-inflammatory drugs (NSAIDs), which are not typically considered antibiotics. Here, we investigate the effects of NSAIDs on bacterial susceptibility to antibiotics and the modulation of bacterial efflux pumps.

**Methodology:**

The activity of 12 NSAID active substances, paracetamol (acetaminophen), and eight relevant medicinal products was analyzed with or without pump inhibitors against 89 strains of Gram-negative rods by determining the MICs. Furthermore, the effects of NSAIDs on the susceptibility of clinical strains to antimicrobial agents with or without PAβN (Phe-Arg-β-naphtylamide) were measured.

**Results:**

The MICs of diclofenac, mefenamic acid, ibuprofen, and naproxen, in the presence of PAβN, were significantly (≥4-fold) reduced, decreasing to 25–1600 mg/L, against the majority of the studied strains. In the case of acetylsalicylic acid only for 5 and 7 out of 12 strains of *P*. *mirabilis* and *E*. *coli*, respectively, a 4-fold increase in susceptibility in the presence of PAβN was observed. The presence of Aspirin resulted in a 4-fold increase in the MIC of ofloxacin against only two strains of *E*. *coli* among 48 tested clinical strains, which included species such as *E*. *coli*, *K*. *pneumoniae*, *P*. *aeruginosa*, and *S*. *maltophilia*. Besides, the medicinal products containing the following NSAIDs, diclofenac, mefenamic acid, ibuprofen, and naproxen, did not cause the decrease of clinical strains’ susceptibility to antibiotics.

**Conclusions:**

The effects of PAβN on the susceptibility of bacteria to NSAIDs indicate that some NSAIDs are substrates for efflux pumps in Gram-negative rods. Morever, Aspirin probably induced efflux-mediated resistance to fluoroquinolones in a few *E*. *coli* strains.

## Introduction

The widespread and frequent prevalence of multidrug (MDR) efflux pumps and the associated multidrug resistance to antibacterial agents among pathogenic bacteria can make the treatment of infectious diseases difficult and ineffective [1–3]. There is an urgent need for new chemical compounds with potent and broad antibacterial activity. Alternatively, looking for effective antibacterial agents among known medicinal products that are routinely used to manage the pathological symptoms of a non-infectious etiology and that are typically considered “non-antibiotics” is a particularly interesting strategy. The potential role of non-antibiotics for treating multidrug-resistant Gram-negative bacteria has been investigated [4–12]. Moreover, the inhibition of MDR efflux pumps by rezerpine, applied in the past as an antipsychotic and antihypertensive drug, has been demonstrated [13].

Efforts have also been undertaken to investigate the antibacterial activity of some compounds belonging to the group of non-steroidal anti-inflammatory drugs (NSAIDs), which are among the most commonly and frequently used medicinal products. The NSAIDs are comprised of several preparations and compounds of different chemical structures, but they all share common properties: analgesic, antipyretic, and anti-inflammatory activity. To date, the best studied NSAID with regards to non-antibiotic activity is diclofenac. It has antimicrobial activity against a broad spectrum of clinical species, including *Escherichia coli* [14,15], *Klebsiella* sp., *Salmonella* sp., *Shigella* sp. and *Vibrio cholerae* [14]. Furthermore, it has been shown that diclofenac inhibits bacterial DNA synthesis [16]. Recently, the mechanism of action of small molecules from the NSAIDs group, such as bromfenac, carprofen, and vedaprofen has been demonstrated [17]. These NSAIDs inhibit the *E*. *coli* DNA polymerase III b subunit which disturbs DNA replication. Targeting the bacterial DNA replication machinery is a validated strategy for producing antibacterial chemotherapeutics like quinolones. In contrast to the fluoroquinolones, the NSAIDs that inhibit DNA replication exhibit weak antibacterial activity [17]. In the case of fluoroquinolones, one of the mechanisms of bacterial resistance is the overexpression of MDR efflux pumps [1,18]. Alternatively, the NSAID salicylate is a known substrate for efflux pumps in *Burkholderia cenocepacia* [19]. It is possible that other NSAIDs are also actively removed from Gram-negative rods by efflux systems and therefore have only poor antimicrobial activity.

Furthermore, direct antimicrobial activity by NSAIDs such as acetylsalicylic acid against *E*. *coli*, *P*. *aeruginosa* [20], and *Helicobacter pylori* [21] has been described. Moreover, it was found that susceptibility of *H*. *pylori* to antibiotics increased in the presence of acetylsalicylic acid [21,22]. Additionally, the activity of ibuprofen and indomethacin against *H*. *pylori* was also observed [23].

It seems that the most important feature of non-antibiotic drugs, besides their therapeutic use, is their ability to inhibit or enhance the activity of some efflux pumps in Gram-negative rods. It is known that some phenothiazines inhibit efflux pumps in Gram-positive bacteria [5]. Alternatively, salicylate, a natural substrate for efflux pumps in *Burkholderia cenocepacia*, can induce efflux-mediated resistance [19]. Salicylate-induced efflux pump expression has also been observed in *E*. *coli* [24], *S*. *enterica* serovar Typhimurium [25], and *C*. *jejuni* [26]. Hence, the potential antibacterial properties and influence of NSAIDs on MDR efflux pump activity are very interesting.

The main goal of this study was to investigate the influence of non-antibiotics from the NSAIDs group on the activity of MDR efflux pumps in Gram-negative bacteria. Modulation of MDR efflux pumps by NSAIDs could modify bacterial susceptibility to antibiotics. The research was performed in two steps:

1. determine the susceptiblility of standard and clinical Gram-negative strains to selected antimicrobial agents and non-antibiotics from the NSAIDs group (active substances and medicinal products) tested in the presence or absence of efflux pump inhibitors (EPIs);
2. investigate of the influence of NSAIDs with or without EPIs on the susceptibility of clinical strains to antimicrobial agents.

We were also interested in finding answers to the following questions:

* Could some NSAIDs be extruded from bacteria by MDR efflux pumps?
* Do NSAIDs influence the activity of efflux pumps and modify bacterial susceptibility to antibiotics?

## Materials and Methods

### Bacterial strains and growth conditions

The following standard strains were used in the study: *E*. *coli* ATCC 25922, NCTC 10538 and NCTC 8196; *Klebsiella pneumoniae* ATCC 13883 and ATCC 700603; *Proteus mirabilis* ATCC 12453; *Proteus vulgaris* ATCC 13315 and NCTC 4635; *Enterobacter cloacae* DSM 6234; *P*. *aeruginosa* ATCC 27853, ATCC 25616, NCTC 6749 and PAO1; *Stenotrophomonas maltophilia* ATCC 13637 and ATCC 12714; *Acinetobacter baumannii* ATCC 19606; and *Burkholderia cepacia* ATCC 25416. These studies also included 72 clinical strains, i.e. a dozen strains of six Gram-negative rod species (*E*. *coli*, *K*. *pneumoniae*, *P*. *mirabilis*, *P*. *aeruginosa*, *S*. *maltophilia*, and *A*. *baumannii*). Clinical strains were isolated from different hospitalized patient samples obtained in Warsaw from 2007 to 2010 and were identified by routine methods using API tests (bioMérieux). All strains were stored at -80°C until analysis. Prior to testing, each strain was sub-cultured twice on TSA (bioMérieux) medium for 24 to 48 h at 30°C to ensure viability.

### Antimicrobial agents, efflux pump inhibitors, NSAID active substances and medicinal products

Three antimicrobial agents: chloramphenicol (Sigma), nalidixic acid (Sigma), and ofloxacin (Sigma), as well as two EPIs: Phe-Arg-β-naphtylamide (PAβN; Sigma) and reserpine (Sigma), were used in the study.

The following 12 NSAID active substance standards, paracetamol (as an alternative analgesic and antipyretic drug), and eight relevant medicinal products were analyzed in this study: diclofenac (Amoli Organics Pvt. Ltd.) and Olfen (50 mg tablets, Mepha), Diclac (25 mg/mL injection, Sandoz GmbH), nimesulide (Polfa Pabianice) and Aulin (100 mg tablets, Medicom International), naproxen (Polfarmex) and Naproxen (500 mg tablets, Hasco-lek), ibuprofen (Ambon Group S.p.A.) and Nurofen (200 mg tablets, Reckitt Benckiser), mefenamic acid and Mefacit (250 mg tablets, Polfa Pabianice), acetylsalicylic acid (Polfa Warszawa) and Aspirin (500 mg tablets, Bayer), paracetamol (R. P. Scherer) and Apap (500 mg tablets, US Pharmacia), piroxicam (USP) and meloxicam (Boehringer Ingelheim Vetmedica GmbH—International). Active substances: mefenamic acid, phenylbutazone, indometacin, metamizole sodium, salicylamide, and salicylic acid, were received from the Medical University of Warsaw. Sodium salicylate was obtained from Sigma.

### Determination of the MICs of antimicrobial agents, NSAID active substances and medicinal products with and without an efflux pump inhibitor

The MIC values of antimicrobial agents as well as NSAID active substances and relevant medicinal products in the presence or absence of EPIs were estimated on Mueller-Hinton II (MH II) agar (Becton Dickinson), using double agent dilutions, according to the CLSI guidelines [27]. Bacterial suspensions at a density 0.5 McFarland units (Densimat; bioMérieux) were diluted 1:10 and 1.5 μL (10^4^ cfu/mL) were applied to the surface of the agar plates. The plates were incubated at 35°C for 18 h. The assay was validated by the MIC determination of selected antimicrobial agents against reference strains (*E*. *coli* ATCC 25922 and *P*. *aeruginosa* ATCC 27853) and comparing the experimental values with the CLSI guidelines [28].

To estimate the MIC values of NSAID medicinal products, tablets were homogenized and then resuspended in the same way as NSAID active substances. The amount of active substances in the obtained material was calculated in comparison to NSAID active substance concentration.

Additionally, the MIC values of the Diclac injection (diclofenac), in the presence or absence of EPIs, were estimated not only on MH II agar, but also in MH II broth (Becton Dickinson), using double agent dilutions, according to the CLSI guidelines [27].

To determine the ability of strains to remove antimicrobial agents or NSAIDs by MDR efflux pumps, the MIC values of antimicrobial agents, NSAID active substances and medicinal products (tablets), with or without the pump inhibitors, PAβN or reserpine, were evaluated. The concentration of both EPIs used was 80 mg/L, as described in our previous study [29]. At least a 4-fold decrease in the MIC values after the addition of PAβN or reserpine was considered significant [29].

### Determination of antimicrobial agent activity in the presence of NSAID medicinal products with and without an efflux pump inhibitor

The influence of NSAID medicinal products, which are actively removed by MDR efflux pumps, on the susceptibility of clinical strains from selected Gram-negative rod species to antimicrobial agents, in the presence or absence of PAβN, was investigated by determining the MIC values of quinolones and chloramphenicol. The MICs of antimicrobial agents were estimated on MH II agar medium.

The selection of medicinal products utilized for this part of the study was based on the results obtained from determining the susceptibility of clinical strains to NSAIDs in the presence of efflux pumps inhibitors. These NSAID medicinal products in tablet form were selected when there was at least a 4-fold decrease in the MIC values of the NSAID in the presence of PAβN, when compared to the MIC values of the NSAID alone. The following concentrations of particular medicinal products were equal to a quarter of the lowest MIC value determined for each group of species that were tested in the presence of PAβN: Olfen (12.5 mg/L—*E*. *coli* and *S*. *maltophilia*, 25 mg/L—*K*. *pneumoniae*, *A*. *baumannii* and *P*. *aeruginosa*, 100 mg/L—*P*. *mirabilis*), Mefecit (12.5 mg/L—*S*. *maltophilia*, 25 mg/L—*E*. *coli*, *K*. *pneumoniae*, *P*. *mirabilis*, *A*. *baumannii* and *P*. *aeruginosa*), Nurofen (100 mg/L—*S*. *maltophilia* and *P*. *aeruginosa*, 200 mg/L—*E*. *coli*, *P*. *mirabilis* and *A*. *baumannii*, 400 mg/L—*K*. *pneumoniae*), Naproxen (100 mg/L—*E*. *coli*, *S*. *maltophilia* and *P*. *aeruginosa*, 200 mg/L—*K*. *pneumoniae*, *P*. *mirabilis* and *A*. *baumannii*), and Aspirin (400 mg/L for all species).

At least a 4-fold change in the MIC value of an antimicrobial agent after the addition of a NSAID medicinal product was considered significant. Additionally, the effect of an NSAID on the susceptibility of clinical strains to antimicrobial agents in the presence of PAβN was analyzed. At least a 4-fold change in the MIC value of an antimicrobial agent in the presence of both the NSAID and PAβN, when compared to the MIC of an antimicrobial agent in the presence of PAβN only, was considered relevant.

### Determination of quinolone activity in the presence of metabolic products of Aspirin with and without an efflux pump inhibitor

The influence of the active substance in Aspirin tablets (acetylsalicylic acid) and the products of its metabolism (sodium salicylate and salicylic acid) on the activity of quinolones (ofloxacin and nalidixic acid), in the presence or absence of PAβN, was analyzed by determining the MIC values of each antimicrobial agent for clinical strains of selected Gram-negative rod species. The MICs of quinolones were estimated in MH II broth as well as MH II agar. The following concentrations were used: 1 mM acetylsalicylic acid, 1 mM sodium salicylate and 1 mM salicylic acid. At least a 4-fold change in the MIC values of quinolones after the addition of salicylate was considered significant.

## Results

### Susceptibility of bacteria to NSAID active substances and medicinal products

The susceptibility of all the investigated bacterial strains to NSAID active substances and paracetamol, as well as the relevant medicinal products, was compared. Only a 2-fold dilution difference between the MIC values of NSAIDS active substances present in standard or tablet form was seldom observed. The content of the active compounds was considered and calculated. Table 1 shows the data on the most potent preparations assessed in the study. The following active substances/relevant medicinal products showed antibacterial activity (MIC ≤3200 mg/L) against the greatest number of tested standard strains: diclofenac (100%), acetylsalicylic acid (100%), salicylamide (76%), ibuprofen (35%), and naproxen (29%). Moreover, diclofenac showed the highest antibacterial activity of all the tested NSAIDs, with a MIC of 200 to 3200 mg/L (Table 1). The remaining substances tested, i.e. indometacin, mefenamic acid, meloxicam, metamizole, phenylbutazone, and piroxican, did not inhibit the growth of the standard strains (MIC >3200 mg/L). Paracetamol, at 3200 mg/L, inhibited the growth of *S*. *maltophilia* ATCC 13637 and ATCC 12714.

**Table 1:** The activity of NSAIDs (active substances and medicinal products) with and without the efflux pump inhibitor PAβN against standard Gram-negative strains.

Strains	MIC (mg/L)						
	dic/Olfen, Diclac^a^ (+PAβN)^c^	mef.a/Mefacit^a^ (+PAβN)^c^	ibup/Nurofen^a^ (+PAβN)^c^	nap/Naproxen^a^ (+PAβN)^c^	nim/Aulin^a^ (+PAβN)^c^	acet/Aspirin^a^ (+PAβN)^c^	salic^b^ (+PAβN)^c^
*E*. *cloacae* DSM 6234	1600 **(50)**	>3200 **(50)**	>3200 **(400)**	>3200 **(400)**	>1600 (>1600)	3200 (1600)	>3200 (3200)
*E*. *coli* ATCC 25922	1600 **(50)**	>3200 **(50)**	3200 **(400)**	3200 **(400)**	>1600 (>1600)	1600 (1600)	1600 (1600)
*E*. *coli* NCTC 10538	1600 **(50)**	>3200 **(50)**	>3200 **(400/800)**	>3200 **(800)**	>1600 (>1600)	3200 (1600)	1600 (1600)
*E*. *coli* NCTC 8196	1600 **(50)**	>3200 **(50)**	>3200 **(400/800)**	>3200 **(800)**	>1600 (>1600)	3200 (1600)	3200 (1600)
*K*. *pneumoniae* ATCC 13883	1600 **(100)**	>3200 **(100)**	>3200 **(800/1600)**	>3200 **(400)**	>1600 (>1600)	3200 (1600)	>3200 (3200)
*K*. *pneumoniae* ATCC 700603	1600 **(200)**	>3200 (>3200)	>3200 (>3200)	>3200 **(800/1600)**	>1600 (>1600)	3200 (1600)	3200 (1600)
*P*. *vulgaris* ATCC 13315	3200 **(200)**	>3200 (>3200)	>3200 **(1600)**	>3200 **(800)**	>1600 (>1600)	3200 (1600)	>3200 (>3200)
*P*. *vulgaris* NCTC 4635	200 (100)	>3200 **(100/200)**	1600 (800)	1600 **(400)**	>1600 (>1600)	1600 (1600)	1600 (1600)
*P*. *mirabilis* ATCC 12453	800 **(200)**	>3200 **(50/100)**	1600 (800)	1600 (800)	>1600 (>1600)	1600 (1600)	>3200 (>3200)
*P*. *aeruginosa* ATCC 27853	1600 **(100)**	>3200 **(100)**	>3200 **(400)**	>3200 **(400)**	>1600 (>1600)	3200 (1600)	1600 (800)
*P*. *aeruginosa* ATCC 25616	1600 **(50)**	>3200 **(50)**	>3200 **(400/800)**	>3200 **(200)**	>1600 (>1600)	3200 (1600)	3200 (1600)
*P*. *aeruginosa* NCTC 6749	1600 **(200)**	>3200 **(50)**	>3200 **(200)**	>3200 **(200)**	>1600 (>1600)	3200 (1600)	3200 (1600)
*P*. *aeruginosa* PAO1	1600 **(200)**	>3200 **(100)**	>3200 **(400)**	>3200 **(400)**	>1600 (>1600)	3200 (1600)	1600 (800)
*A*. *baumannii* ATCC 19606	1600 **(200)**	>3200 **(200)**	3200 **(800)**	>3200 **(800)**	>1600 (>1600)	3200 (1600)	1600 (800)
*B*. *cepacia* ATCC 25416	800 (800)	>3200 (>3200)	>3200 **(1600)**	>3200 **(1600)**	>1600 (>1600)	3200 (1600)	1600 (1600)
*S*. *maltophilia* ATCC 13637	800 **(25)**	>3200 **(25)**	3200 **(100)**	3200 **(100)**	>1600 **(100)**	1600 (800)	1600 (800)
*S*. *maltophilia* ATCC 12714	1600 **(50)**	>3200 **(50)**	1600 **(200)**	3200 **(200)**	>1600 **(200)**	1600 (1600)	1600 (1600)

dic, diclofenac; mef.a, mefenamic acid; ibup, ibuprofen; nap, naproxen; nim, nimesulide; acet, acetylsalicylic acid; salic, salicylamide; PAβN, Phe-Arg-β-naphthylamide.

^a^When a difference between the MIC values of the NSAID active substance and medicinal product with and without PAβN was observed, both MICs of the NSAID active substance and its medicinal product are presented.

^b^Salicylamide was studied only as the active substance.

^c^At least a 4-fold decrease in the MIC of NSAID active substances and medicinal products in the presence of PAβN, when compared to the MIC values of NSAIDs without PAβN, is indicated in boldface.

Four NSAID active substances (diclofenac, ibuprofen, naproxen, and acetylsalicylic acid), paracetamol, and six relevant medicinal products were selected for the further investigation of NSAIDs activity against clinical strains. The characteristics of the sensitivity of 72 selected clinical strains belonging to six Gram-negative rod species to antibacterial compounds, such as quinolones and chloramphenicol, which are substrates of MDR efflux pumps, are presented in Table 2. A significant decrease in the MIC values for at least one of the three tested antibiotics was observed in the presence of the EPI PAβN in all of the clinical isolates used in this study.

**Table 2:** Susceptibility of clinical strains of Gram-negative rods to selected antimicrobial agents in the presence or absence of PAβN.

Bacteria (No. of isolates)	Antimicrobial agent^a^	MICs range (mg/L)^b^		No. of strains^c^
		MH	MH+PAβN	
*E*. *coli* (n = 12)	**ofloxacin**	1–64	0.125–8	**12**
	nalidixic acid	1024->2048	64–256	12
	chloramphenicol	8->256	1–64	12
*K*. *pneumoniae* (n = 12)	**ofloxacin**	8–32	0.5–4	**12**
	nalidixic acid	4->2048	1–512	12
	chloramphenicol	8->256	1–64	11
*P*. *mirabilis* (n = 12)	ofloxacin	2–256	2–256	0
	nalidixic acid	>2048	256–512	12
	**chloramphenicol**	8–512	4–128	**7**
*S*. *maltophilia* (n = 12)	ofloxacin	1–8	1–8	1
	**nalidixic acid**	8–32	2–4	**12**
	chloramphenicol	4–64	1–32	1
*A*. *baumannii* (n = 12)	ofloxacin	8–64	4–64	3
	nalidixic acid	1024->2048	128–256	12
	**chloramphenicol**	32–256	8–64	**12**
*P*. *aeruginosa* (n = 12)	**ofloxacin**	2–128	0.063–2	**12**
	nalidixic acid	64–2048	1–32	12
	chloramphenicol	8–256	1–4	12

PAβN, efflux pump inhibitor Phe-Arg-β-naphthylamide; MH, Mueller-Hinton II medium.

^a^Antimicrobial agents used to study the influence of NSAIDs on antibacterial activity against different bacteria species are indicated in boldface.

^b^The correctness of the assay was verified by determining the MIC of antimicrobial agents necessary to inhibit growth of reference strains (*E*. *coli* ATCC 25922 and *P*. *aeruginosa* ATCC 27853) and comparing with CLSI guidelines: ofloxacin MIC was 0.03 mg/L for *E*. *coli* and 2 mg/L for *P*. *aeruginosa*, nalidixic acid MIC was 2 mg/L for *E*. *coli* and 512 mg/L for *P*. *aeruginosa*, chloramphenicol MIC was 2 mg/L for *E*. *coli*.

^c^Number of strains with at least a 4-fold decrease in MICs in the presence of PAβN.

Generally, diclofenac and the relevant medicinal products (Olfen and Diclac) were active against all 72 studied clinical strains, with MIC values ranging from 800 to 3200 mg/L (Table 3). Unlike the bacteria from the *Enterobacteriaceae* family, the non-fermentative Gram-negative rods were also sensitive to acetylsalicylic acid (and Aspirin). Furthermore, other NSAIDs like ibuprofen, naproxen, and their relevant medicinal products were not active against clinical strains of *E*. *coli*, *K*. *pneumoniae*, *P*. *mirabilis*, *P*. *aeruginosa*, and *A*. *baumannii* (Table 3).

**Table 3:** The activity of NSAIDs and paracetamol (active substances and medicinal products) against clinical isolates of *Enterobacteriaceae* and non-fermentative Gram-negative rods.

Bacteria (No. of strains)	NSAID substance (Medicinal product)	No. of isolates with MIC values			
		800 mg/L	1600 mg/L	3200 mg/L	>3200 mg/L
*E*. *coli* (n = 12)	diclofenac (Olfen, Diclac)	0 (0)	12 (12)	0 (0)	0 (0)
*K*. *pneumoniae* (n = 12)	diclofenac (Olfen, Diclac)	0 (0)	12 (12)	0 (0)	0 (0)
*P*. *mirabilis* (n = 12)	diclofenac (Olfen, Diclac)	0 (0)	3 (3)	9 (9)	0 (0)
*P*. *aeruginosa* (n = 12)	diclofenac (Olfen, Diclac)	0 (0)	12 (12)	0 (0)	0 (0)
	acetylsalicylic acid (Aspirin)^a^	0 (0)	6 (0)	6 (12)	0 (0)
*S*. *maltophilia* (n = 12)	diclofenac (Olfen, Diclac)	4 (4)	8 (8)	0 (0)	0 (0)
	ibuprofen (Nurofen)	1 (1)	10 (10)	1 (1)	0 (0)
	naproxen (Naproxen)^a^	0 (0)	11 (0)	1 (11)	0 (1)
	acetylsalicylic acid (Aspirin)^a^	1 (0)	8 (4)	3 (8)	0 (0)
	paracetamol (Apap)^b^	0 (0)	0 (0)	12 (12)	0 (0)
*A*. *baumannii* (n = 12)	diclofenac (Olfen, Diclac)	0 (0)	12 (12)	0 (0)	0 (0)
	acetylsalicylic acid (Aspirin)	0 (0)	1 (1)	11 (11)	0 (0)

^a^Only in case of *P*. *aeruginosa* and *S*. *maltophilia* strains was a 2-fold dilution difference between the MIC values of the active substance and the medicinal product observed.

^b^Paracetamol and its relevant medicinal product, Apap tablet, were used in this study as an alternative analgesic and antipyretic drug.

### The effect of the efflux pump inhibitors on the susceptibility of bacteria to NSAIDs

Thesusceptibility of 17 standard strains to 12 NSAID active substances, paracetamol and eight relevant medicinal products in the presence of two efflux pump inhibitors was tested. For the study carried out on clinical strains, six NSAID active substances (diclofenac, mefenamic acid, ibuprofen, naproxen, nimesulide, and acetylsalicylic acid) and their relevant medicinal products were selected. Except for acetylsalicylic acid, they all showed increased activity (decreases in MIC values of at least a 4-fold) against standard strains in the presence of PAβN, when compared to its absence. Neither PAβN nor reserpine (80 mg/L) inhibited the growth of any of the tested strains.

There were no differences greater than 2-fold between the MIC values of the active substances present in the standard and the tablet, when tested in the presence of efflux pump inhibitors. Between the two studied pump inhibitors, only PAβN affected the susceptibility of the majority of Gram-negative bacteria to NSAIDs.

The effect of PAβN on the MIC values of NSAIDs for standard and clinical strains is presented in Table 1 and in Table 4, respectively. Among the NSAIDs, the MIC values of naproxen, diclofenac, mefenamic acid, and ibuprofen (as active substances and as medicinal products) in the presence of PAβN were significantly (≥4-fold) reduced against the majority of the standard strains (from 16 to 14 out of 17; 94–82%, respectively) (Table 1). Furthermore, a potent (≥32-fold) increase in bacterial susceptibility to mefenamic acid (for 13 standard strains) and diclofenac (for 7 strains) was observed.

**Table 4:** Effects of PAβN on the MIC values of tested NSAIDs against clinical isolates of *Enterobacteriaceae* and non-fermentative Gram-negative rods.

Bacteria (No. of strains)	NSAID substance (Medicinal product)	No. of isolates with indicated fold reduction in NSAID MICs in the presence of PAβN				
		≥ 4-fold	≥ 8-fold	≥ 16-fold	≥ 32-fold	≥ 64-fold
*E*. *coli* (n = 12)	diclofenac (Olfen, Diclac)	12 (12)	12 (12)	12 (12)	12 (12)	0 (0)
	mefenamic acid (Mefacit)	12 (12)	12 (12)	12 (12)	12 (12)	12 (12)
	ibuprofen (Nurofen)	12 (12)	0 (0)	0 (0)	0 (0)	0 (0)
	naproxen (Naproxen)	12 (12)	0 (0)	0 (0)	0 (0)	0 (0)
	acetylsalicylic acid (Aspirin)	7 (7)	0 (0)	0 (0)	0 (0)	0 (0)
*K*. *pneumoniae* (n = 12)	diclofenac (Olfen, Diclac)	12 (12)	12 (12)	11 (11)	2 (1)	0 (0)
	mefenamic acid (Mefacit)	12 (12)	12 (12)	12 (12)	12 (12)	4 (4)
	ibuprofen (Nurofen)	12 (12)	0 (0)	0 (0)	0 (0)	0 (0)
	naproxen (Naproxen)	12 (12)	3 (3)	0 (0)	0 (0)	0 (0)
*P*. *mirabilis* (n = 12)	diclofenac (Olfen, Diclac)	12 (12)	8 (8)	2 (2)	0 (0)	0 (0)
	mefenamic acid (Mefacit)	4 (4)	4 (4)	4 (4)	4 (4)	0 (0)
	ibuprofen (Nurofen)	5 (4)	0 (0)	0 (0)	0 (0)	0 (0)
	naproxen (Naproxen)	12 (12)	4 (4)	0 (0)	0 (0)	0 (0)
	acetylsalicylic acid (Aspiryn)	5 (5)	0 (0)	0 (0)	0 (0)	0 (0)
*P*. *aeruginosa* (n = 12)	diclofenac (Olfen, Diclac)	12 (12)	12 (12)	10 (10)	2 (3)	0 (0)
	mefenamic acid (Mefacit)	9 (9)	9 (9)	9 (8)	8 (8)	3 (2)
	ibuprofen (Nurofen)	12 (12)	12 (11)	5 (4)	1 (0)	0 (0)
	naproxen (Naproxen)	12 (12)	12 (12)	12 (11)	2 (2)	0 (0)
*S*. *maltophilia* (n = 12)	diclofenac (Olfen, Diclac)	12 (12)	12 (12)	12 (2)	5 (5)	0 (0)
	mefenamic acid (Mefacit)	12 (12)	12 (12)	12 (12)	12 (12)	12 (12)
	ibuprofen (Nurofen)	12 (12)	9 (9)	1 (1)	0 (0)	0 (0)
	naproxen (Naproxen)	12 (12)	10 (10)	1 (1)	0 (0)	0 (0)
*A*. *baumannii* (n = 12)	diclofenac (Olfen, Diclac)	12 (12)	12 (12)	1 (1)	1 (1)	0 (0)
	mefenamic acid (Mefacit)	12 (12)	12 (12)	12 (12)	12 (12)	5 (5)
	ibuprofen (Nurofen)	12 (12)	8 (7)	0 (0)	0 (0)	0 (0)
	naproxen (Naproxen)	12 (12)	3 (2)	0 (0)	0 (0)	0 (0)

PAβN, efflux pump inhibitor Phe-Arg-β-naphthylamide.

When assessing the susceptibility of standard strains to acetylsalicylic acid, indometacin, meloxicam, metamizole, phenylbutazone, piroxicam, salicylamide, and paracetamol, with or without pump inhibitors, we did not observe at least a 4-fold decrease in the MIC values of these substances.

Importantly, with four of six analyzed NSAIDs, i.e. diclofenac, mefenamic acid, ibuprofen, and naproxen (active substances and medicinal products), a significant reduction (≥4-fold) in these NSAID MICs was observed in the presence of the PAβN for the clinical strains of all six studied species of Gram-negative rods. The magnitude of the reductions in the NSAID MICs for each group of rods is presented in Table 4. The highest increase in bacterial susceptibility to NSAIDs in the presence of PAβN was observed for diclofenac (Olfen and Diclac) and mefenamic acid (Mefacit) in all or a majority of the isolates of *S*. *maltophilia* (MICs 25–100 mg/L and MIC 100 mg/L, respectively), *E*. *coli* (MIC 50 mg/L and MIC 100 mg/L, respectively), *K*. *pneumoniae* (MICs 50–200 mg/L and MICs 100–200 mg/L, respectively), *A*. *baumannii* (MICs 50–200 mg/L and MICs 100–200 mg/L, respectively), *P*. *aeruginosa* (MICs 50–200 mg/L and MICs 100–400 mg/L, respectively), and *P*. *mirabilis* (MICs 200–400 mg/L and MIC 200 mg/L, respectively).

Furthermore, a significantly lower increase in susceptibility to ibuprofen (Nurofen) and naproxen (Naproxen) for all the tested clinical isolates was observed. The MIC values of these two NSAIDs decreased to 400–800 mg/L in the case of *S*. *maltophilia* strains and to 800–1600 mg/L for other tested Gram-negative rods.

Interestingly, some of the tested *E*. *coli* and *P*. *mirabilis* clinical strains also showed a 4-fold increase in susceptibility to acetylsalicylic acid (Aspirin) in the presence of PAβN. On the other hand, at least a 4-fold decrease in the MIC values of nimesulide (Aulin) was not observed against any clinical strain.

### The effect of NSAIDs on the susceptibility of clinical strains to antimicrobial agents with and without PAβN

The susceptibility of clinical strains to antimicrobial agents actively extruded by efflux pumps in the presence of NSAIDs was studied. Antimicrobial agents for each group of species and the NSAID medicinal products were selected based on data obtained in the previous investigations (Tables 2 and 4). At least a 4-fold decrease in the MIC values of the selected NSAID medicinal products, including Olfen, Mefacit, Nurofen, Naproxen, and Aspirin, was previously observed in the presence of the PAβN (Table 4).

Among the five studied NSAID medical products, only Aspirin tablets (containing acetylsalicylic acid) affected the MICs of ofloxacin against two strains of *E*. *coli* (Table 5). The presence of Aspirin in concentration below 1/4 MIC resulted in 4-fold increased MIC values of this fluoroquinolone.

**Table 5:** Effect of NSAID medicinal products on the MICs of different antimicrobial agents tested with and without PAβN against clinical Gram-negative rods (12 isolates of each species).

Bacteria	Agent MICs range [mg/L]	No. of strains with ≥4-fold decrease (increase^a^) in antimicrobial agent MICs in the presence of NSAID ± PAβN						
		At+ PAβN	At+Nf^b^	At+Nf+ PAβN^c^	At+Nx^b^	At+Nx+ PAβN^c^	At+As^b^	At+As+ PAβN^c^
*E*. *coli*	ofloxacin (1–64)	12	0	0	0	0	(**2**^a^)	0
*K*. *pneumoniae*	ofloxacin (8–32)	12	0	0	0	0	0	0
*P*. *mirabilis*	chloramphenicol (8–512)	7	0	0	0	0	0	0
*P*. *aeruginosa*	ofloxacin (2–128)	12	0	0	0	0	0	0
*S*. *maltophilia*	nalidixic acid (8–32)	12	0	0	0	0	0	0
*A*. *baumannii*	chloramphenicol (32–256)	12	0	**9**	0	**11**	0	0

At, antimicrobial agent; PAβN, efflux pump inhibitor; Nf, Nurofen (containing ibuprofen); Nx, Naproxen (containing naproxen); As, Aspirin (containing acetylsalicylic acid).

^a^At least a 4-fold increase in the MIC of each antimicrobial agent in the presence of NSAID^b^ ± PAβN^c^, when compared to the MIC^b^ of the antimicrobial agent ± PAβN^c^.

^b^At least a 4-fold decrease in the MIC of each antimicrobial agent in the presence of NSAID, when compared to the MIC of the antimicrobial agent alone.

^c^At least a 4-fold decrease in the MIC of each antimicrobial agent in the presence of NSAID and PAβN, when compared to the MIC of the antimicrobial agent in the presence of only PAβN.

Interestingly, in the case of the majority of *A*. *baumannii* isolates, at least a 4-fold decrease in chloramphenicol MIC values in the presence of Nurofen or Naproxen and PAβN was observed, as compared to antimicrobial agent MICs in the presence of PAβN only (Table 5). Other investigated medicinal products, like Olfen and Mefacit, did not affect the susceptibility of studied clinical strains of Gram-negative rods to ofloxacin, chloramphenicol, and nalidixic acid.

### The effect of salicylate on the susceptibility of clinical strains to quinolones with and without PAβN

Among the 48 tested clinical strains belonging to four Gram-negative rod species (*E*. *coli*, *K*. *pneumoniae*, *P*. *aeruginosa*, and *S*. *maltophilia*), only in case of two *E*. *coli* strains was a 4-fold increase in the ofloxacin MIC observed in the presence of 1 mM salicylic acid; for one of them, this was also observed in the presence of 1 mM sodium salicylate (Table 6). The MIC values of ofloxacin increased from 16 mg/L to 64 mg/L for one strain, and from 32 mg/L to 128 mg/L for another strain. Additionally, a non-significant (only 2-fold) increase in the ofloxacin MIC was obtained in the presence of 1 mM acetylsalicylic acid, 1 mM sodium salicylate, and 1 mM salicylic acid in case of 6, 7, and 8 out of 12 tested *E*. *coli* strains, respectively. Moreover, only a 2-fold increase in the nalidixic acid MIC was also observed in the presence of 1 mM of acetylsalicylic acid and its metabolites in the case of some *S*. *maltophilia* strains (Table 6).

**Table 6:** Effects of acetylsalicylic acid and its metabolites on the susceptibility of Gram-negative clinical strains (12 isolates of each species) to quinolones in the presence or absence of PAβN.

Bacteria	Agent MICs range [mg/L]	No. of strains with ≥4-fold (only 2-fold) increase in antimicrobial agent MICs in the presence of NSAID ± PAβN						
		At+ PAβN	At+Ac^a^	At+Ac+ PAβN^b^	At+SNa^a^	At+SNa+ PAβN^b^	At+S^a^	At+S+ PAβN^b^
*E*. *coli*	ofloxacin (1–64)	12 (0)	0 (6)	0 (5)	1 (7)	0 (9)	2 (8)	0 (12)
*K*. *pneumoniae*	ofloxacin (8–32)	12 (0)	0 (0)	0 (3)	0 (0)	0 (1)	0 (0)	0 (3)
*P*. *aeruginosa*	ofloxacin (2–128)	12 (0)	0 (0)	0 (7)	0 (0)	0 (7)	0 (0)	0 (11)
*S*. *maltophilia*	ofloxacin (1–8)	0 (0)	0 (0)	0 (0)	0 (0)	0 (0)	0 (0)	0 (0)
	nalidixic acid (8–32)	12 (0)	0 (2)	0 (0)	0 (7)	0 (0)	0 (7)	0 (0)

At, antimicrobial agent; PAβN, efflux pump inhibitor; Ac, acetylsalicylic acid 1 mM; SNa, sodium salicylate 1 mM; S, salicylic acid 1 mM.

^a^At least a 4-fold (only 2-fold) increase in the MIC of the antimicrobial agent in the presence of NSAID, when compared to the MIC of antimicrobial agent alone.

^b^At least a 4-fold (only 2-fold) increase in the MIC of the antimicrobial agent in the presence of NSAID and PAβN, when compared to the MIC of the antimicrobial agent in the presence of only PAβN.

## Discussion

One method for developing new therapeutic options for the treatment of multidrug-resistant Gram-negative rods is to analyze the potential role of non-antibiotics [4]. Unfortunately, the majority of non-antibiotic agents belonging to different therapeutic groups, such as antianaplastics, antiarrhythmics, antihypertensives, antidepressives, anticonvulsatives, spasmolytics, and anti-inflammatory drugs, show only marginal direct antibacterial activity (MIC ≥3000 mg/L) [30,31]. Currently, diclofenac [14,15] and acetylsalicylic acid [20,21] are the only NSAIDs to show some antibacterial activity against Gram-negative rods. Additionally, ibuprofen and indomethacin have activity against *H*. *pylori* [23]. However, the MIC values of diclofenac [14,15] and acetylsalicylic acid [20] against Gram-negative rods were not determined in accordance with current CLSI recommendations; therefore, these data could not be compared with our results.

In this study, low MIC values (below 1600 mg/L) in the case of diclofenac, ibuprofen, acetylsalicylic acid, and relevant medicinal products were observed only for the four standard strains of Gram-negative rods and several *S*. *maltophilia* clinical isolates.

On the other hand, it is known that MDR efflux pumps from the RND family play a significant role in the resistance of Gram-negative rods to several antibiotics from different chemical groups [1–3]. Furthermore, RND efflux systems can remove some disinfectant agents, aromatic hydrocarbons, acriflavine, rhodamine 6G, vanadium, crystal violet, and ethidium bromide from bacteria [3]. One could speculate that MDR efflux pumps are responsible for the lack of or weak NSAID activity against Gram-negative bacteria. A basic *in vitro* phenotypic screening test of antibiotic removal from bacteria by MDR efflux pumps consists of measuring the changes in the MICs of the antibiotic in the absence and presence of efflux pump inhibitors [32–34]. The most commonly used MDR efflux pump inhibitor for these studies is phenylalanyl arginyl β-naphthylamide (PAβN or MC-207,110) [1,13,29,34–39]. This compound was established as the first inhibitor of RND transporters in Gram-negative rods [34,40]. PAβN potently inhibits the efflux systems from the Mex family in *P*. *aeruginosa* (especially MexAB-OprM) [34,39,41] and also inhibits the AcrAB-TolC efflux system in *E*. *coli* [37,41,42]. The occurrence of the AcrAB efflux pump has also been described in other species of the *Enterobacteriaceae* family, e.g. *Enterobacter cloacae* [43], *Klebsiella pneumoniae* [44], *Salmonella enterica* Serovar typhymurium [45], and *Proteus mirabilis* [46].

In this study, analyzing NSAID activity against bacteria, it was shown that the MIC values of naproxen, diclofenac, mefenamic acid, and ibuprofen (as active substances and medicinal products) in the presence of PAβN were significantly reduced against the majority of the standard and clinical strains of Gram-negative rods. Interestingly, some of the clinical *E*. *coli* and *P*. *mirabilis* strains also showed a 4-fold increase in susceptibility to acetylsalicylic acid (Aspirin) in the presence of PAβN. This is the first observation regarding the effect of an efflux pump inhibitor on the activity of NSAIDs against a broad spectrum of Gram-negative bacteria. These NSAIDs are probably substrates for RND efflux systems. Previously, it was described that salicylic acid is a substrate for the CeoAB-OpcM efflux system in *B*. *cenocepacia* [19]. In this study, it was shown for the first time that NSAID active substanes (such as mefenamic acid, diclofenac, ibuprofen, and naproxen) were actively removed, probably by the efflux pumps present in *Enterobacteriaceae* as well as in non-fermentative Gram-negative rods. Moreover, these results indicate that the presence of efflux pumps may be one reason for the weak activity of some NSAIDs against Gram-negative rods.

Most patients with bacterial infections suffer from fever and pain that require complex treatment with antibiotics, antipyretics, and analgesics. NSAIDs are the most common drugs used to relieve symptoms of diseases caused by bacteria. The question arises as to whether NSAIDs may influence the activity of bacterial efflux pumps and thus modify bacterial susceptibility to antibiotics. Knowledge of the effects of NSAIDs on antibiotic treatment in the context of drug interactions with bacterial efflux pumps is limited. Currently, salicylic acid is the only known NSAID which can induce efflux-mediated resistance in some Gram-negative rods like *E*. *coli* [24], *S*. *enterica* serovar Typhimurium [25], and *B*. *cenocepacia* [19].

Our results indicate that one of the studied NSAID medicinal products, Aspirin tablets (containing acetylsalicylic acid), affected the antibiotic susceptibility of only a few *E*. *coli* strains of the 72 tested clinical Gram-negative isolates. The presence of Aspirin at a concentration of 400 mg/L (2.2 mM) resulted in a 4-fold increase in the MIC values of ofloxacin for only two of 12 tested *E*. *coli* strains. The effect of salicylate on the antibiotic resistance of Gram-negative rods has already been described [24,47,48]. However, those studies were conducted using laboratory strains and mutants, not clinical isolates. Acetylsalicylic acid and salicylate increased the resistance of *E*. *coli* to multiple antibiotics such as quinolones (nalidixic acid and norfloxacin), tetracycline, chloramphenicol, and ampicillin, but not to aminoglycosides [24,47,48]. Salicylate-induced antibiotic resistance in *E*. *coli* is due to increased transcription of the *marRAB* operon, encoding the global regulator MarA [24]. This enhanced production of the MarA protein increases the transcription of the *acrAB* operon, which leads to overexpression of the multidrug AcrAB-TolC efflux system. Alternatively, this global regulator reduces the production of the outer membrane porin OmpF, thus limiting the influx of some antibiotics into the bacterium [49]. Moreover, in *E*. *coli*, two other MDR efflux pumps, EmrAB [48,50] and EmrKY [48,51], are also affected by salicylate. Importantly, salicylate-induced *marRAB* expression is concentration-dependent [24]. At concentrations between 0.01–0.1 mM of salicylate, the *mar* promoter was not induced. Only at concentrations above 0.5 mM of salicylate was the expression of *marRAB* observed [24]. The observation of salicylate-induced antibiotic resistance of *E*. *coli* in the presence of 5 mM salicylate has limited therapeutic value [24,47].

Contrary to the data published for *E*. *coli* and *S*. *enterica* serovar Typhimurium, salicylate had no significant effect on the expression of the *emrRCABsm* operon (encoding EmrCABsm efflux pump from MFS family) in *S*. *maltophilia* [52] as well as two operons, *adeFGH* and *adeIJK* (encoding AdeFGH and AdeIJK efflux pumps from RND family), in *A*. *baumannii* [53]. Moreover, at 2.5–4 mM salicylate, the expression of the *adeABC* operon (encoding the AdeABC RND efflux pump) in *A*. *baumannii* was reduced by 2.5-fold, which did not affect the susceptibility level of this strain to ciprofloxacin, gentamicin, and ceftriaxone [53].

A high concentration of acetylsalicylic acid in the human body can be reached only transiently because it is rapidly hydrolyzed to salicylic acid in the stomach and in the liver [54]. Salicylic acid and salicylate are the principal metabolites of acetylsalicylic acid [25]. A plasma level of Aspirin, 20–100 mg/L (0.1–0.55 mM) is recommended for analgesia and 150–300 mg/L (0.83–1.67 mM) for an anti-inflammatory effect. In the case of salicylate, it is assumed that a therapeutic level is up to 1.8 mM in the plasma [21]. It has also been shown that a low concentration of salicylate (0.1–0.01 mM), which is commonly achieved by therapeutic doses of Aspirin, selectively blocks COX-2 transcription in humans [55,56]. A relatively high concentration of Aspirin (2 mM) is used to treat chronic inflammatory diseases such as rheumatoid arthritis [57,58]. It was originally thought that acetylsalicylic acid and salicylate at concentrations of 5 mM or higher were toxic to humans [56,59]. However, it has recently been demonstrated that plasma levels greater than 2.2 mM are potentially toxic in patients chronically treated with salicylate [21]. Considering the therapeutic concentrations of acetylsalicylic acid and its metabolites in plasma, we used a 1 mM concentration of acetylsalicylic acid, sodium salicylate, and salicylic acid. A similar concentration was previously used to test the effects of salicylate on the antibiotic susceptibility of *Campylobacter jejuni* [26]. The presence of salicylate resulted in only a moderate (2-fold) increase in the MIC of ciprofloxacin in *C*. *jejuni* NCTC 11168. Moreover, salicylate did not affect the MICs of other antibiotics like nalidixic acid, florfenicol, clindamycin, azithromycin, rifampicin, cefotaxime, and aminoglycosides. However, the presence of salicylate induced (by 3-fold) the expression of *cmeABC*, which encodes for a MDR efflux system in *C*. *jejuni* [26]. In our study, only two *E*. *coli* strains exhibited a 4-fold increase in the MICs of ofloxacin in the presence of Aspirin tablets, the active substance, and its metabolites.

Based on our results and previous studies on the effect of salicylate on antibiotic resistance and the induction of efflux pump expression in Gram-negative rods (*E*. *coli* [24,47,48], *S*. *enterica* serovar Typhimurium [25,48], *B*. *cenocepacia* [19]), and *C*. *jejuni* [26]), we suggest the following explanation. The presence of Aspirin and its metabolites at therapeutic concentrations (1–2 mM) only slightly increases the MIC of ofloxacin against clinical isolates of *E*. *coli*, despite an earlier study that described the effect of salicylate on the induction of the AcrAB-TolC efflux system [24]. Alternatively, resistance to antibiotics, including fluoroquinolones, in Gram-negative bacteria is determined by the presence of various independent mechanisms such as point mutations in the *gyr* and *par* genes, overexpression of different MDR efflux pumps, and dysregulation of influx [1,18,60,61]. These factors impact the observed results obtained using clinical isolates and laboratory strains.

In conclusion, two NSAID substances, diclofenac and acetylsalicylic acid, showed weak direct antimicrobial activity against standard strains and clinical isolates, while the three other NSAIDs (ibuprofen, naproxen, and salicylamide) were only active against some standard strains. Moreover, it was shown for the first time that NSAIDs (such as mefenamic acid, diclofenac, ibuprofen, naproxen, and acetylsalicylic acid) and relevant NSAID medicinal products (Mefacit, Olfen, Diclac, Nurofen, Naproxen, and Aspirin) are substrates for the efflux pumps in some Gram-negative rods. Importantly, among the investigated NSAID medicinal products, only Aspirin induced efflux-mediated resistance to ofloxacin in two strains of *E*. *coli* out of 48 tested clinical strains belonging to the species *E*. *coli*, *K*. *pneumoniae*, *P*. *aeruginosa*, and *S*. *maltophilia*.

Antibacterial therapy for patients also taking NSAIDs containing acetylsalicylic acid should be carefully monitored, because the administered antibiotics could be removed from the bacteria by the efflux pumps stimulated by Aspirin.

In light of this study, NSAIDs containing active substances which are not extruded by MDR pumps (indometacin, meloxicam, metamizole, phenylbutazone, piroxicam) and paracetamol as well as compounds which are efflux pump system substrates, but do not affect antibiotic resistance (diclofenac, mefenamic acid, ibuprofen and naproxen) can be safely used as antipyretic or analgesic substances during antibacterial treatment.
